# Methylmercury Interactions With Gut Microbiota and Potential Modulation of Neurogenic Niches in the Brain

**DOI:** 10.3389/fnins.2020.576543

**Published:** 2020-11-03

**Authors:** Daniel V. Pinto, Ramon S. Raposo, Gabriella A. Matos, Jacqueline I. Alvarez-Leite, João O. Malva, Reinaldo B. Oriá

**Affiliations:** ^1^Laboratory of Tissue Healing, Ontogeny and Nutrition, Department of Morphology, School of Medicine, Institute of Biomedicine, Federal University of Ceara, Fortaleza, Brazil; ^2^Experimental Biology Core, University of Fortaleza, Fortaleza, Brazil; ^3^Department of Biochemistry and Immunology, Institute of Biological Sciences, Federal University of Minas Gerais, Belo Horizonte, Brazil; ^4^Center for Innovative Biomedicine and Biotechnology (CIBB), Faculty of Medicine, Institute of Pharmacology and Experimental Therapeutics, Institute for Clinical and Biomedical Research (iCBR), University of Coimbra, Coimbra, Portugal

**Keywords:** methylmercury, neurogenesis, brain, intestinal microbiota, neurodegenerative diseases, gut dysbiosis

## Introduction

Mercury (Hg) is a well-recognized biohazard for the nervous system. Methylmercury (MeHg) is an organic methylated form of Hg, highly toxic to humans, targeting the brain, as MeHg is rapidly absorbed, and easily reaches and crosses the blood-brain barrier (Takahashi et al., 2017). Neurological symptoms may vary from acute motor and visual effects to marked behavioral and psychiatric alterations. At higher neurotoxic levels, MeHg can lead to irreversible coma and, ultimately, death. It has been highlighted that MeHg long-term and low-grade toxicity may be associated with neurodegenerative disorders and perhaps a direct causality for Alzheimer's disease (Siblerud et al., 2019).

Although MeHg harmful effects to the brain have been thoroughly documented in the literature, such as increased oxidative stress and mitochondrial dysfunction, halted glutamate uptake by astrocytes and overt glutamate excitotoxicity, and activation of neuronal apoptosis cascades (Antunes dos Santos et al., 2016), less is known how MeHg affects the hippocampal neurogenic niche.

Hence, in this opinion paper, we summarize up-to-date literature addressing MeHg effects on the intestinal microbiota, a key player influencing MeHg bioavailability and MeHg induction of intestinal dysbiosis (and vice-versa), and related intricate mechanisms during homeostasis and disease states. In addition, we discuss possible ways how MeHg may affect hippocampal neurogenesis and the potential lasting consequences for brain neurodegeneration.

## Intestinal Microbiota is Affected by MeHg

In mammals, the intestinal microbiota is first acquired either by contact with maternal skin (if a cesarean labor) or directly maternal microbiota transfer (if by vaginal labor) immediately after birth (Shao et al., 2019) and through breast milk feeding (Pannaraj et al., 2017). The intestinal microbiota diversity is highly dynamic in the first years of post-natal life, until it reaches its “adulthood-like” characteristics in early childhood (Oriá et al., 2018). The first 2-years of life is also a time window for important early post-natal brain plasticity events, such as active synaptogenesis, myelination, and neurogenesis (Lebel and Deoni, 2018). Moreover, enteric infections may prevail early in life when individuals are exposed to enteric pathogens and pathogenic bacteria, especially in unprivileged settings of the developing world, where poor sanitation and hygiene much often occurs (Oriá et al., 2016). In the first post- natal years, the brain is particular vulnerable to environmental toxicants, as well (Rodier, 1995).

The intestinal absorption of MeHg may, indeed, be modulated by the intestinal microbiota and by a healthy (intact) intestinal barrier function. Impaired intestinal barrier function has been associated with increased MeHg intestinal absorption (Zhao et al., 2020), altered blood-brain barrier, and disrupted VEGF signaling (Takahashi et al., 2017).

A “leaky gut” may markedly increase circulating LPS, trigger peripheral inflammation and oxidative stress that can reach the central nervous system, leading to brain neuroinflammation, with increased microglia priming and activation (García-Domínguez et al., 2018). Circulating LPS levels in peripheral blood have been associated with a reduction in neurogenesis and mild cognitive deficit in experimentally Alzheimer's disease model (Valero et al., 2014).

MeHg-driven intestinal injury may be protected by orally-given *Lactobacillus*, a known bacteria that can generate short- chain fatty acids (SCFAs) in the large intestine (Jiang et al., 2018). SCFAs are important for microglia maturation and function (Wenzel et al., 2020) and reduced SCFA production by the gut microbiota has been related to neurodegenerative diseases (Zhang et al., 2017). Of note, fecal samples from human APOE4 carriers (individuals at higher risk to develop Alzheimer's disease) are significantly less enriched in *Ruminococcacea* family of anaerobes associated with fiber fermentation and production of SCFAs (Tran et al., 2019).

One form of modulation is the demethylation of MeHg into inorganic elements by the intestinal microbiota, which reduces Hg solubility in the tissue and hence bioavailability to the brain. The upregulation in *Peptococcaceae* family in the rat gut has been found to promptly demethylate MeHg and increase fecal excretion of Hg elements (Lin et al., 2020). Fecal excretion is one important way to eliminate Hg and prevent MeHg intoxication (Clarkson and Magos, 2006). Two-genes clusters, hgcA and hgcB, are required for mercury methylation by gut bacteria (Parks et al., 2013). Several hgcAB-encoding bacterial strains may affect gut net Hg bacterial methylation (Zhang et al., 2019).

Another microbiota-directly related event is the modulation of bacterial siderophores, that can bind to Hg molecules, acting as chelators, forming insoluble complexes and facilitating their excretion in the feces (Schalk et al., 2011). Such effect may modulate the bioavailability of other metals such as iron and zinc, brain trophic key nutrients, that may also regulate intestinal bacterial overgrowth (Lopez and Skaar, 2018).

Oral administration of HgCl2 to female mice can also induce alterations in the intestinal microbiota homeostasis increasing the *Firmicutes/Bacteroidetes* ratio (Ruan et al., 2019). This alteration in *Firmicutes/Bacteroidetes* ratio is also seen with the use of antibiotics (Indiani et al., 2018), immunosuppressive agent or even intestinal chronic diseases (Bhat et al., 2017). This ratio has been used as a proxy of an unhealthy gut, being positively correlated with gut dysbiosis and obesity (Leocádio et al., 2020). Furthermore, it has been associated with aging and Alzheimer's disease (Hoffman et al., 2017).

Studies with MeHg intoxication in 8-week old mice have shown reduced *Firmicutes* and increased *Bacteroidetes* numbers compared with non-intoxicated controls (Zhang et al., 2019). In contrast to the metagenomic data from Zhang and colleagues, Lie et al. studies have found that MeHg-orally exposed young rats (10 μg/Kg) showed altered intestinal microbiota with reduced relative abundance of *Bacteroidetes* and *Protobacteria* and increases in *Firmicutes* (Lin et al., 2020). This discrepancy between both studies may be due to different animals and age groups used.

The increase ratio of *Bacteroidetes* and *Firmicutes* in young animals may be relevant to hippocampal development and neurogenesis, as the first week of life in rodents are an important time window of brain plasticity. Studies with germ-free mice suggest that a time window early in life is when gut microbes are more modulatory in disturbing hippocampal neurogenesis (Cerdó et al., 2020).

Another relevant alteration in the intestinal microbiota, that has been reported, is the increase in *Akkermansia* from stools of MeHg-exposed pregnant humans (Rothenberg et al., 2016). These bacteria have been associated with reduced intestinal barrier function and loss of the intestinal mucus layer (Yoshihara et al., 2020). Impairment of the intestinal mucus layer increases the risk for gut dysbiosis, intestinal inflammation, and gut-to-blood pathogenic bacteria translocation (Bergstrom et al., 2010), which may be facilitated by MeHg-induced intestinal injury (Zhao et al., 2020).

## MeHg Effects on Hippocampal Neurogenesis. Potential Modulation By the Gut Microbiota?

The hippocampus is critical for memory and learning. These processes rely on complex and intricate neuronal circuitry, involving dentate gyrus granular, CA3 and CA1 pyramidal cell synapses (excitatory trisynaptic neuronal circuit) (Beer et al., 2018). The former neurons are continued renewed at the hippocampal subgranular zone (SGZ) neurogenic niche, which harbors neural stem cells (NSC). The hippocampal neurogenic niche is more active in childhood and may be affected by early-life adverse events with lasting effects (Cohen et al., 2016).

NSC undergoes three maturational stages before reaching full maturity. Stage 1: containing radial-like NSC (GFAP, nestin, and SOX2 positive cells); stage 2, containing transition cells/progenitor cells type 2a (GFAP, nestin, and SOX2 positive cells) and type 2b (nestin, DCX, Neuro-D, and Prox 1 positive cells) and stage 3 (DCX, Neuro-D, and Prox1 positive cells). After stage 3, NSCs reach neuronal maturity by the extension of dendritic and axon processes and functional synaptic activity into neural circuitry (calretin, NeuN, Neuro-D, and Prox1 positive cells) (Kempermann et al., 2015).

MeHg effects on hippocampal neurogenesis have been mostly studied in the first week of post-natal life in animal models when there is active hippocampal neurogenesis. MeHg exposure reduces 21% of the total volume of DNA in the hippocampus, 16% of the total positive Brdu cells in the granular layer and 50% of cells in the hilus, 14 days after the 5.0 μg/g s.c. injection on the post-natal day (PN) 7 (Falluel-Morel et al., 2007). Kim et al. (2019), also found a 57% reduction in the number of positive Ki-67 cells in the dentate gyrus in 7-week-old Sprague-Dawley rats, 35 days after 5.0 μg/g MeHg injection (Kim et al., 2019).

A reduction of 22% of hilus cells and 27% of granular layer cells, without affecting CA1 to CA3 hippocampal fields, after the MeHg challenge, has been found in mice, suggesting that MeHg preferentially affects SGZ's NSC (Sokolowski et al., 2013). Besides, primary cultures of NSC following exposure to MeHg showed reduced expression of NADH dehydrogenase and cytochrome B (Bose et al., 2012), supporting a pro-apoptotic effect of MeHg in NSC cells. A double immunolabeling of dentate gyrus SOX2 and cleaved caspase 3 cells induced by MeHg intoxication further suggests the direct target of MeHg to early hippocampal NSCs (Falluel-Morel et al., 2007).

Falluel-Morel work also documented a decrease in the extracellular signaling of ERK1/2, required for the transition from G1 to S phase and inhibition of Bmi-1 gene expression, responsible for controlling the self-renewal potential of NSC. Genes linked to cell senescence have also been shown to be altered by MeHg, such as increased expression levels of HP1-γ and HMGA-1 (Falluel-Morel et al., 2007). Long-term antibiotic therapy that disrupts intestinal microbiota homeostasis with increased number of conditionally pathogenic enterobacteria, *E*. *coli, Clostridium, Staphylococcus* spp., and hemolytic bacteria, could affect redox-sensitive transcription factor HIF1α and ERK1/2 MAP kinase and intestinal barrier function (Holota et al., 2019).

Interestingly, early life stress, such as social isolation, can impact neurogenesis and the intestinal microbiota. Socially isolated rats early in life show significantly fewer BrdU/NeuN positive cells in the dentate gyrus than controls and altered microbiota composition with increases in *Actinobacteria* and decreases in the class *Clostridia* (Dunphy-Doherty et al., 2018). It would be interesting to know whether these alterations may be further affected by early- life mercury intoxication.

One possible crosstalk pathway between intestinal microbes and the brain may involve the vagal nerve. Vagotomy has been implicated in impaired hippocampal neurogenesis and BDNF levels (O'Leary et al., 2018). Importantly, mercury intoxication has been implicated in disturbed vagal nerve function (Simões et al., 2016).

The better understanding how the MeHg-altered gut microbiota affects the hippocampal neurogenic niche, by tracking neurogenic biomarkers during NSC maturation in critical time windows of brain development, is in most need for novel therapeutic strategies to ameliorate these deleterious effects that may have lasting consequences for human health.

## Conclusion and Perspectives

This opinion paper discussed the role of the intestinal microbiota on MeHg neurointoxication with potential consequences for the hippocampal neurogenic niche. Novel breakthrough findings are much supportive of human neurogenesis even in elderly (Boldrini et al., 2018), which was not universally accepted (Sorrells et al., 2018). Activity of NSCs and renewal of hippocampal neurons even in adulthood may be a protective/preventive factor against neurodegenerative diseases. New available neuroimaging tools to study the human brain neurogenesis may shed light to MeHg deleteriously effects on dynamic processes occurring in the hippocampal neurogenic niche during lifespan.

Accumulating evidence implies the gut-brain axis as a pathway for MeHg harmful neurotoxic effects and a potential factor for later neurodegenerative disorders. The MeHg may induce a hormesis-related neuronal toxicity. Hormesis is an important redox dependent aging-associated neurodegenerative/ neuroprotective issue (Calabrese et al., 2010). The use of antioxidants, such as plant polyphenols (Calabrese et al., 2010; Leri et al., 2020) and protective nutrients (Oria et al., 2020) may be beneficial in reducing the MeHg-driven neuroinflammatory state and associated cell death with the interplay of the intestinal microbiota. Further research is warranted to elucidate the fine molecular mechanisms and NSC biomarkers during lifelong hippocampal neurogenesis and how they are affected by MeHg. Such findings may contribute to health policies in highly endemic MeHg intoxication and poor sanitation settings.

## Author Contributions

All authors have read and approved the manuscript. All authors have equally contributed to the opinion paper.

## Conflict of Interest

The authors declare that the research was conducted in the absence of any commercial or financial relationships that could be construed as a potential conflict of interest.
